# The Impact of Coinfections and Their Simultaneous Transmission on Antigenic Diversity and Epidemic Cycling of Infectious Diseases

**DOI:** 10.1155/2014/375862

**Published:** 2014-06-22

**Authors:** Xu-Sheng Zhang, Ke-Fei Cao

**Affiliations:** ^1^Department of Statistics, Modelling and Economics, Centre for Infectious Disease Surveillance and Control, Public Health England, 61 Colindale Avenue, London NW9 5EQ, UK; ^2^Medical Research Council Centre for Outbreak Analysis and Modelling, Department of Infectious Disease Epidemiology, Imperial College Faculty of Medicine, Norfolk Place, London W2 1PG, UK; ^3^Centre for Nonlinear Complex Systems, Department of Physics, Yunnan University, Kunming, Yunnan 650091, China

## Abstract

Epidemic cycling in human infectious diseases is common; however, its underlying mechanisms have been poorly understood. Much effort has been made to search for external mechanisms. Multiple strains of an infectious agent were usually observed and coinfections were frequent; further, empirical evidence indicates the simultaneous transmission of coinfections. To explore intrinsic mechanisms for epidemic cycling, in this study we consider a multistrain Susceptible-Infected-Recovered-Susceptible epidemic model by including coinfections and simultaneous transmission. We show that coinfections and their simultaneous transmission widen the parameter range for coexistence and coinfections become popular when strains enhance each other and the immunity wanes quickly. However, the total prevalence is nearly independent of these characteristics and approximated by that of one-strain model. With sufficient simultaneous transmission and antigenic diversity, cyclical epidemics can be generated even when strains interfere with each other by reducing infectivity. This indicates that strain interactions within coinfections and cross-immunity during subsequent infection provide a possible intrinsic mechanism for epidemic cycling.

## 1. Introduction

Infectious diseases cause huge morbidity and mortality in human populations and remain as a dramatic threat to public health [1, 2]. The incidence of many infectious diseases varies periodically. Cyclical infections of humans range from childhood infections such as measles, pertussis, mumps, diphtheria, varicella, and scarlet fever [3] to faecal-oral infections such as cholera and rotavirus and vector-borne diseases such as malaria and dengue fever [4, 5]. For example, seasonal flu develops as an epidemic during winter in temperate regions but it remains at very low levels during summer [6] while pertussis has 3-4-year cycles [7]. Despite the pervasive nature of oscillation, its underlying mechanisms are not well understood. From detailed analyses of empirical data, several distinct mechanisms have been proposed [4, 6, 8]: survival of disease pathogen outside host; host behaviour; seasonal changes in host susceptibility and immune defence and in vector abundance. Overall, those mechanisms have accounted only for the variation caused by external factors. Mechanically, they can be explained by a standard Susceptible-Infected-Recovered (SIR) or Susceptible-Infected-Recovered-Susceptible (SIRS) transmission model that was embedded with a seasonal transmission rate (i.e., the so-called “seasonal forcing”) [4, 5, 8–12]. However, these models cannot provide a reasonable explanation for, say, a quickly globally spreading pattern of influenza [13] and require unrealistically high reproduction rates for the observational seasonal patterns in influenza [14]. Moreover, Grassly et al. [15] show that it is the intrinsic factors (e.g., immunity following the recovery from syphilis infection), rather than the external factors (e.g., changes in human sexual behaviour), that cause the 8–11-year cycles in syphilis incidence from the 1960s to the 1980s in the United States cities. Other mechanisms were also sought [16]. A single-strain standard SIR model that was not embedded with explicit seasonal forcing cannot generate sustained oscillations in incidence unless other complications are included. For example, variants of the standard SIR model such as cyclic models of SIRS and SEIRS type with a large time delay in the removed class, models with nonlinear force of infection, models with variable population size and disease-related deaths [17], or models including an isolated class generate sustained oscillations albeit under unrealistic region of parameter space [18]. However, it has been recognised that, without consideration of the intrinsic aspects of infectious diseases such as interactions between strains of an infectious agent, the understanding of epidemic cycling cannot be complete [10, 19] or is impossible [20].

Due to the high mutation rate and other genetic processes (such as recombination and reassortment), pathogens can quickly generate a lot of different genotypes. The striking fact of infectious agents is their antigenically diverse strains that are cocirculating within host populations [21–26] and concurrent infections with multiple strains [27–31]. Polymorphic pathogens such as dengue virus [22], malaria parasite* Plasmodium falciparum* [21], influenza virus [23, 24], and* Streptococcus pneumoniae* [26] are responsible for enormous health burdens of human populations [26, 32, 33]. Understanding the underlying mechanisms of antigenic diversity and cyclical epidemics will provide theoretical bases for their evolution under selective pressure such as the replacement of strains and the emergence of resistance strains under pharmacological control measures [25]. In practice, this will also be supplied with the basis for the design of effective control strategies such as vaccination [34].

In general, once two different pathogen strains infect the same host individuals, they will interact with each other [32]. The interaction can be classified into direct interactions of pathogen genes or gene products (such as recombination in bacterium* Streptococcus* and reassortment in influenza A viruses) and indirect interactions that result from alteration in the host environment such as immunological responses [35]. The indirect interactions may be synergistic, neutral, or antagonistic in view of changes in transmissibility and virulence [2, 33, 35–37]. For example, epidemiological evidence suggests that primary infection with one strain of dengue virus can enhance transmission of subsequent infection with another strain [38–40]. Further, the strains present in coinfections can transmit simultaneously and separately [29, 41, 42]. The direct observation of transmission process from one to another person is hard even for single transmission, let alone simultaneous transmission of multiple strains. To our knowledge, the one direct evidence of simultaneous transmission (or cotransmission) came from the field study during 2009 pandemic influenza by Liu et al. [29]. Cross-immunity between different subtypes of influenza has been observed [43–46]. Strong cross-immunity has been observed among antigenic drift variants of the same influenza subtype; for example, the first wave of the 1918 (H1N1) pandemic provided up to 94% protection against clinical illness during the second wave [47]. Cross-immunity was also found between different strains of other infectious agents [48, 49]. These characteristics surely affect pathogen diversity and dynamical behaviour of polymorphic pathogens. Though a large body of epidemiological researches are devoted to understanding the mechanisms of host single-strain pathogen dynamics, the increased evidence has gathered that the characteristics of polymorphic pathogen causing infections prove deviant from the predictions from one-strain models. For example, estimation of characteristics of transmission dynamics of polymorphic malaria* Plasmodium falciparum* that is based on one-strain epidemic model is misleading [50]; further, coinfections may imply that the survivorship of strains is not necessarily maximized by maximizing the basic reproductive number [51].

Transmission consequences of coinfection and superinfection have received a great deal of interest over the last 30 years [37, 52–58]. Theoretical modelling and empirical analyses show that pathogen interactions could have profound implications for the pathogen communities [51, 59–61]. For example, it has been shown that cocirculation of different diseases and cross-immunity [59] and antibody-dependent enhancement in transmission [38, 39, 62] can induce cyclical and chaotic epidemics. Cross-immunity alone within a two-strain SIR or SIRS model cannot generate sustained oscillation [12, 63–65] unless other factors such as age-dependent survival [53, 57] or isolation [66] are included. Following the traditional assumption that individuals, while being infected with one strain, are temporarily not susceptible to the other [52, 53, 62, 67], most of all these models consider only subsequent infection (i.e., individuals that recovered from primary infection with one strain were further infected by others). Even in those that included coinfections, different strains are not assumed to simultaneously transmit [12, 58, 61, 67, 68].

Recent empirical evidence has challenged the traditional assumption and indicated the concurrent infections during the infectious period of primary infection [29, 32, 41, 69, 70]. Due to technical difficulty and complexity involved in distinguishing multiple strains, multiple-strain infections are surely underestimated as argued by Balmer and Tanner [32]. The available empirical studies suggest that the occurrence of multiple strains was significantly more common than expected by chance. For example, Vaccarella et al. [70] found that the observed-to-expected ratio for concurrent infections with two human papillomavirus (HPV) types was 1.16 and for 3–7 types was 1.04. This implies that prior infection by one strain may facilitate infection by the other and strain interactions within coinfections can be synergism [42, 61]. For influenza that spreads from person-to-person through aerosols of droplets nuclei, for example, infection with one strain may increase the aerosolisation and hence the transmissibility of coinfecting other strains [71]. It is hence necessary to explore the possible role that strain interactions within concurrent infections might play in maintaining pathogen diversity and generating oscillatory diseases.

In this study we focus on the intrinsic factors of cyclical epidemics within a multistrain SIRS model by assuming that coinfections can directly transmit from people to people but at a low rate in relation to single transmission. We consider the indirect strain interactions that do not produce novel strains (cf. [59, 72]). In view of evidence of strong cross-immunity among strains (e.g., [47]), we assume a special situation where the patients recovered from infection will become completely cross-immune against any subsequent infections during their protective immunity periods. We thus exclude subsequent infection (cf. [38, 62, 67]). For concurrent infection of strains of a polymorphic pathogen, we have not found any informative data about the impact of order of infection and therefore ignore the impact of infection passage and superinfection [37, 42, 54]. We investigate how the interplay of complete cross-immunity during subsequent infection and strain interactions within coinfection affects antigenic diversity structure and generates cyclical epidemics.

## 2. Models and Methods

We consider a human population in which there are *n* cocirculating strains and every individual is capable of being simultaneously infected by at most *m*  (≤*n*) strains. For convenience, we denote the coinfection of multiplicity *l* as *i*_1_ *i*_2_ ⋯ *i*_*l*_, here *i*_1_, *i*_2_,…, *i*_*l*_ ∈ *H* ≡ {1,2, 3,…, *n*} and *i*_1_ < *i*_2_ < ⋯<*i*_*l*_ representing the strains present in the coinfection, which is independent of order of such infections. Let *I*_*l*,*i*_1_⋯*i*_*l*__ denote the fraction of individuals that carry the coinfection *i*_1_ *i*_2_ ⋯ *i*_*l*_. In this study we define the multiplicity of strains in a coinfection as the within-in host antigenic diversity and the number (*n*) of cocirculating strains in the host population as the antigenic diversity of the population. We consider a SIRS epidemic model by assuming the coinfection *i*_1_ *i*_2_ ⋯ *i*_*l*_ can simultaneously transmit among individuals at rate *β*_*l*,*i*_1_⋯*i*_*l*__; the coinfection simultaneously progresses to the recovered at rate *γ*_*l*,*i*_1_⋯*i*_*l*__ (cf. [34, 73]); strain interactions within coinfection occur via the change in infectivity (cf. [34]). Infections are acquired through a mass action term with the force of infection for the coinfection *i*_1_ *i*_2_ ⋯ *i*_*l*_ given by

(1) $$\begin{array}{c} \Lambda_{l,j_{1}\cdots j_{l}}=\beta_{l,j_{1}\cdots j_{l}}\left[I_{l,j_{1}\cdots j_{l}}+\overset{m}{\underset{v=l+1}{\sum }}\phi_{v,l}\sum_{\left\{j_{1},\ldots ,j_{l}\}\subseteq \{k_{1},\ldots ,k_{v}\right\}}I_{v,k_{1}\cdots k_{v}}\right]. \end{array}$$

Here *ϕ*_*v*,*l*_ represents the factor that *l* strains transmit simultaneously from a patient coinfected with *v*  (>*l*) strains, which reflects the strain interaction within coinfection. If strains interfere with each other, *ϕ*_*v*,*l*_ < 1; otherwise, *ϕ*_*v*,*l*_ > 1 implies enhancement in infectivity of strains. The factor *ϕ*_*v*,*l*_ here is distinct from that proposed in dengue virus models (e.g., [38, 62]) where it reflects the change in transmissibility during secondary infection.

Further we assume that strain interaction also occurs via the change in susceptibility: once recovered from primary infection, patients become completely immune against any strain during the immunity period (1/*σ*), which is assumed to be the same for people recovered from different infections (cf. [51, 61]). This assumption neglects the complicated history of infection and thus largely simplifies the system (cf. [51, 63, 67, 74]). Further we assume that the birth to the susceptible and the loss of population by death are introduced through the same constant rate *μ*, therefore a constant population size. These assumptions lead to a system of coupled differential equations

(2) dSdt=−∑v=1m∑{k1,…,kv}Λv,k1⋯kvS+μ(1−S)   +σ(1−S−∑l=1m∑i1…il∈HIl,i1⋯il),dIl,i1⋯ildt =∑v=1l∑{j1,…,jv}∪{jv+1,…,jl}={i1,…,il}Λv,j1⋯jvIl−v,jv+1⋯jl  −(∑v=1m−l∑{j1,…,jv}∩{i1⋯il}=ØΛv,j1⋯jv)Il,i1⋯il  −(γl,i1⋯il+μ)Il,i1⋯il.

Here *Ø* is used to stand for empty set. In the above equations, *l* = 1,…, *m*, and *S*  ( = *I*_0_) represents the fraction of the susceptible. For coinfection of multiplicity *l*, there are *n*_*l*_ = *n*!/*l*!(*n* − *l*)! different combinations. A very large population is assumed to ignore the stochasticity.

The system at an arbitrary number of strains is intractable. We simplify the problem to investigate only the symmetric multistrain system where all strains share the same epidemiological properties: *β*_1,*i*_ = *β*_1_ and *γ*_1,*i*_ = *γ*_1_,  *i* = 1,…, *n* (cf. [51, 67]). Further, strain interactions are identical and depend only on the multiplicity of coinfections: *β*_2,*ij*_ = *β*_2_,…, *β*_*m*,*i*_1_*i*_2_⋯*i*_*m*__ = *β*_*m*_,  *ϕ*_*j*,*l*_ = *ϕ*_*l*_ for *j* > *l* and *γ*_2,*ij*_ = *γ*_2_,…, *γ*_*m*,*i*_1_*i*_2_⋯*i*_*m*__ = *γ*_*m*_. At the equilibrium of such symmetric system the fraction of individuals that were coinfected with *m* strains is the same among different combinations; for example, *I*_1,*i*__1_ = *I*_1_,…, *I*_*m*,*i*_1_*i*_2_⋯*i*_*m*__ = *I*_*m*_, *i*_1_,…, *i*_*m*_ ∈ *H*. The model system reduces to that of 1 + *m* equations

(3) dSdt=−∑i=1m(ni)ΛiS+μ(1−S)+σ(1−S−∑l=1mnlIl),dIldt=∑i=1l(li)ΛiIl−i−∑j=1m−l(n−lj)ΛjIl−(γl+μ)Il,                      l=1,…,m,

with the force of infection,

(4) Λl=βl[Il+ϕl∑j=l+1m(n−lj−l)Ij],            l=1,…,m.

The prevalence of coinfections of multiplicity *l* is *p*_*l*_ = *n*_*l*_ *I*_*l*_ and the total prevalence is *p* = ∑_*l*=1_^*m*^ *p*_*l*_. For further simplicity we assume that all the efficiencies of partial transmission from coinfections are the same (i.e., *ϕ*_1_ = *ϕ*_2_ = ⋯ = *ϕ*_*m*_ = *ϕ*) and the infectious periods of all infections are the same (i.e., 1/*γ*_1_ = 1/*γ*_2_ = ⋯ = 1/*γ*_*m*_ = 1/*γ*).

## 3. Results

Though analytical results are hard to obtain for the general model system, they can be obtained for a two-strain model which was given in Appendix. Otherwise, our results are mainly based on the numerical integrations of (3) and (4). We first illustrate the invasion conditions for a pathogen strain to the host population in which other strains were established and then demonstrate how coinfections affect the antigenic diversity structure and facilitate the emergence of cyclical epidemics.

### 3.1. Dynamics of Invasion and Coexistence of Multiple Strains

Consider a situation where a host population was already at equilibrium with *n*_*e*_ endemic strains, each of which has a basic reproductive number *R*_0_ = *β*_1_/(*μ* + *γ*). What is the condition for a new indistinguishable strain to invade into the population? In the case of *n*_*e*_ = 1, as shown in Appendix, it is that the efficiency of partial transmission from coinfections (*ϕ*) and the simultaneous transmission rate of double infections (*β*_2_) satisfy the inequality

(5) $$\begin{array}{c} \phi \geq \phi_{s}\equiv 0.5-\frac{\beta_{2}}{2\beta_{1}}. \end{array}$$

This approximated relationship holds for pathogens that have a much shorter infectious period than the duration of immunity such as influenza virus.

Though condition (5) was analytically obtained for the system of two strains, numerical simulations confirm it as a good approximation for multistrain systems (see Table 1). Results show that when the efficiency of partial transmission from coinfections is less than the critical value (*ϕ*_*s*_), only a single strain persists stably. Otherwise, a new indistinguishable strain can successfully invade and coexist with the strains in the previous endemic. That is, once condition (5) is satisfied, arbitrary number of strains can be maintained. Table 1 also indicates that simultaneous transmissions of triple and quadruple infections have a very weak impact on the invasion and pathogen diversity. This implies that, with simultaneous transmission of double infections, pathogen diversity can increase even under fairly strong interference between strains (i.e., *ϕ*_*s*_ is much smaller than the unit) (cf. [73]). The critical value (*ϕ*_*s*_) is 50% in the absence of simultaneous transmission of double infections; with simultaneous transmission (*β*_2_), the critical value (*ϕ*_*s*_) further reduces as predicted in (5) (Table 1). Therefore, given that the basic reproductive number of each strain is larger than unit, equilibrium dynamics fall into two classes depending on the efficiency of partial transmission from coinfections and simultaneous transmission: stable single-strain persistence and multistrain persistence with stable diversity (cf. [67]). Hence it is the strain interaction within coinfection (i.e., *β*_2_ and *ϕ*) that changes the traditional competitive exclusion [67, 73] and facilitates the increase in antigenic diversity.

### 3.2. Structure of Antigenic Diversity

**Figure 1:**
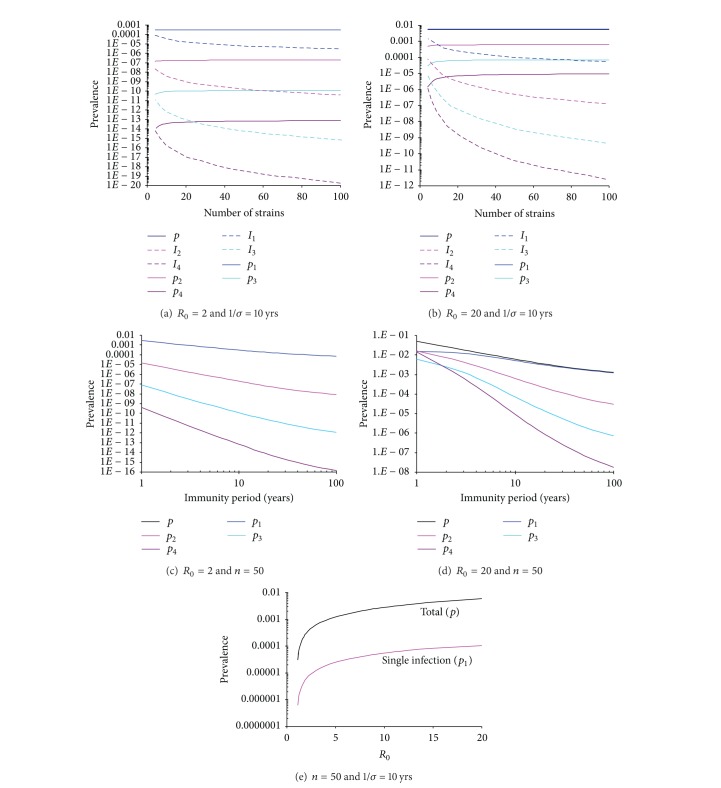
Influence of the number of strains, the immunity period, and the basic reproductive number on the antigenic diversity structure. Model parameters: transmission rate: *β*_1_ = 1.0 day^−1^ and *β*_*l*_ = 0,  *l* ≥ 2,  *ϕ* = 1, and life span (1/*μ*) = 70 years. In panels (a) and (c) infectious period = 2 days and *R*_0_ = 2.0; in panels (b) and (d) infectious period = 20 days and *R*_0_ = 20.0. In panels (a) and (b) the total prevalence overlaps with the prevalence of single infections, implying most infections are single. In panel (e) the total prevalence *p* is well approximated by (6). Note that in panel (d) the prevalence *p*_4_ exceeds *p*_3_ when immunity period is around one year. This is the consequence of the assumption made in the example that the most complex coinfections are of multiplicity 4 so quadruple infections will not be further infected to become other more complex coinfections and hence its prevalence accumulated. The same phenomena also occur in Figure 3.

Under the traditional circumstance where only one strain can transmit (i.e., *β*_1_ > 0 but *β*_*l*_ = 0 for *l* > 1), the antigenic diversity structure is shown in Figure 1. It shows that as the antigenic diversity (*l*) of coinfections increases, the proportion of coinfections of the same *l* strains (i.e., *I*_*l*_) decrease as 1/*n*_*l*_; however, their prevalence (i.e., *p*_*l*_) remains roughly the same. The reduction in *I*_*l*_ with multiplicity *l* is so remarkable that single infections are the most common infections (Figures 1(a) and 1(b)). When transmissibility is relatively low, triple and quadruple infections are prohibited (Figures 1(a) and 1(c)). However, with a high transmissibility, high diverse infections can safely survive (Figures 1(b) and 1(d)). Nevertheless, the total prevalence of all different forms of infections is nearly independent of the number of cocirculating strains and approximated as in the one-strain model system

(6) $$\begin{array}{c} p=\overset{m}{\underset{l=1}{\sum }}p_{l}\approx \frac{\sigma +\mu }{\sigma +\mu +\gamma }\left(1-\frac{1}{R_{0}}\right) \end{array}$$

(see Figure 1(e)). This approximation can be simply confirmed from (3) and (4) under the situation where coinfections are rare. Ignoring coinfections, (3) and (4) reduce to that of one-strain SIR model with the prevalence of one strain replaced by total prevalence of *n* strains (cf. [67]). The derivation of approximation (6) for a special situation of two strains is given in Appendix.

**Figure 2:**
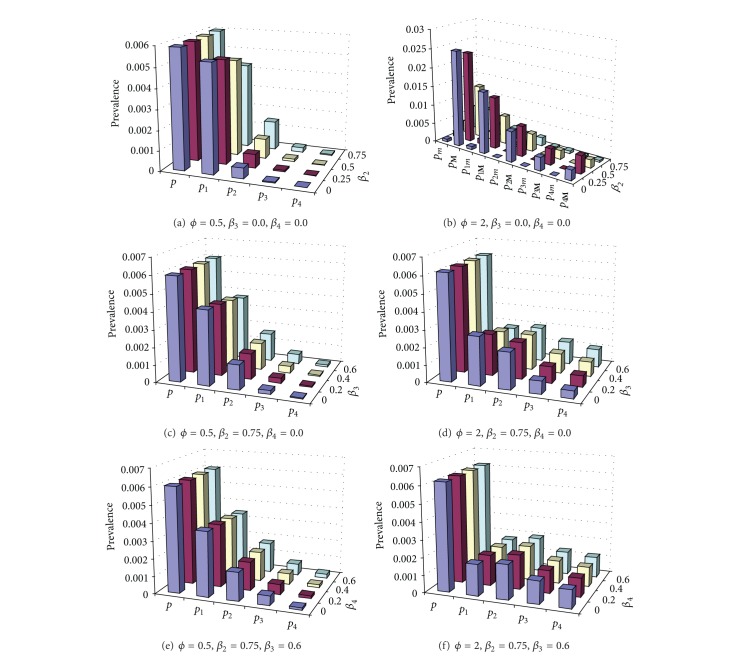
Influence of simultaneous transmission of coinfections and efficiency of partial transmission from coinfections on the prevalence of single infections and coinfections. The effects of each simultaneous transmission rate (*β*_*l*_) of coinfections of multiplicity are shown: panels (a) and (b) *l* = 2; (c) and (d) *l* = 3; and (e) and (f) *l* = 4 while others remain fixed. Two efficiencies of partial transmission from coinfections are shown: *ϕ* = 0.5 (panels (a), (c), and (e)) and =2 (panels (b), (d), and (f)). Model parameter values: immunity period (1/*σ*) = 10 years, transmission rate of single infection (*β*_1_) = 1.0 day^−1^, and infectious period (1/*γ*) = 20 days; hence *R*_0_ = 20; life span (1/*μ*) = 70 years, *n* = 10, and *m* = 4. Note that within panel (b) cyclical epidemics occur when *β*_2_ = 0, 0.25, 0.5 day^−1^ with interepidemic periods around 420 days. The maximum (with subscript *M*) and minimum (with subscript *m*) prevalence are shown.

**Figure 3:**
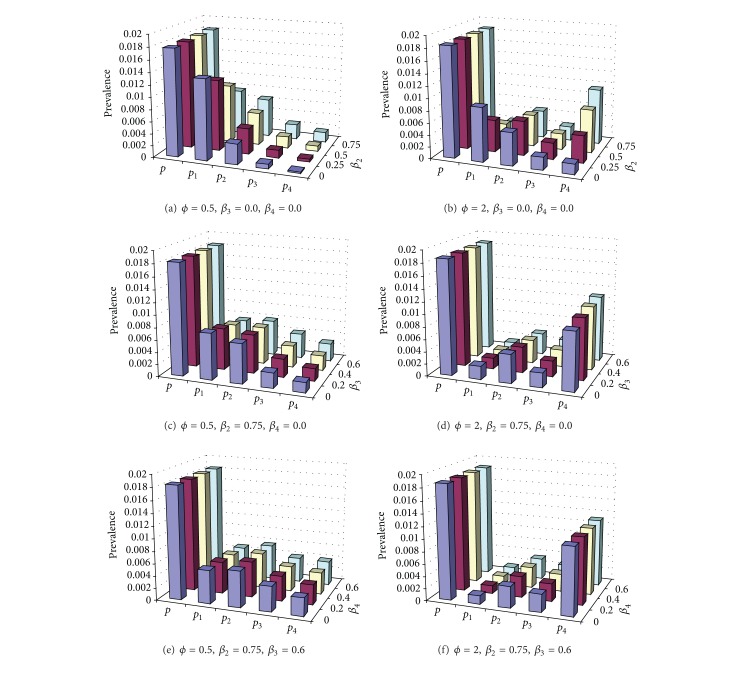
As in Figure 2 except immunity period = 3 years. Under this short immunity period, no cyclical epidemics appear within the ranges of parameters investigated.

The influences of simultaneous transmission of coinfections are shown in Figures 2 and 3 under two different immunity periods. Increasing *β*_2_ increases the prevalence of coinfections but reduces single infections when strains interfere with each other (*ϕ* < 1) (Figures 2(a) and 3(a)). However, if strains interact to enhance the infectivity within coinfections (*ϕ* > 1), cyclical epidemics may emerge when immunity lasts longer (Figure 2(b)) or the prevalence of highly diverse coinfections increases substantially while single infections decrease greatly (Figure 3(b)). However, the impact of simultaneous transmission of more complicated coinfections is much weaker such that *β*_4_ hardly have any effect (Figures 2(e), 2(f), 3(e), and 3(f)). Under the circumstance of *β*_2_ > *β*_3_ > *β*_4_, the total prevalence is dominated by single infections while the triple infections or higher complex coinfections increase but still remain at very low levels if the immunity period is long (cf.  Figure 1(d)) and *ϕ* < 1 (Figures 2(a), 2(c), 2(e), and 3(a)). However, when strains facilitate each other to enhance their infectivity (*ϕ* > 1), single infections cannot dominate (Figures 2(b), 2(d), and 2(f)). Furthermore, in the situation of short immunity period, complicated infections such as double, triple, and quadruple infections become more popular than single infections (Figures 3(b), 3(d), and 3(f)). This indicates that decreasing the duration of immunity can increase coinfection and stabilize the coexistence (cf. Figures 2(b) and 3(b); see also Figure 5(a)). When the coexistence is steady, the total prevalence (*p*) slightly increases with these characteristics. Given all others being the same, increasing *β*_2_ from 0 to (*β*_1_ = )  1 only increases *p* less than 3% while increasing *β*_3_ from 0 to 0.75 and increasing *β*_4_ from 0 to 0.6 only increase *p* by 0.9% and 0.3%, respectively.

The interaction between strains is also reflected in the efficiency of partial transmission from coinfection (*ϕ*). When *ϕ* is not strong (≤1), single infections dominate (Figure 1). When *ϕ* is strong, given all others being the same, coinfections such as double and triple coinfections become common and predominate (Figure 3) and may also induce cyclical epidemics (Figure 2(b)). Nevertheless, as *ϕ* increases from 0 to 2, coinfections predominate but the total prevalence *p* only increases about 5%. Overall, the results cannot exceed 106% of (6) for all the steady scenarios examined in Figures 2 and 3. These observations are also supported by the results listed in Table 2. This indicates that (6) is a good approximation for the total prevalence of steady coexistence. Thus strain interactions (i.e., *β*_2_ and *ϕ*) determine the structure of antigenic diversity but cannot much alter the total prevalence.

### 3.3. Cyclic Epidemics

**Figure 4:**
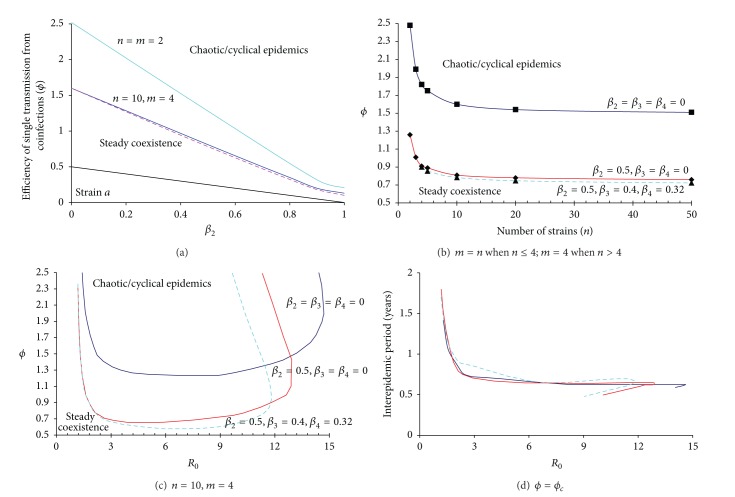
Impact of simultaneous transmission of coinfections and antigenic diversity on the critical value of the efficiency of partial transmission from coinfections (*ϕ*_*c*_) for cyclic epidemics. Other model parameter values are 1/*σ* = 3 years, *β*_1_ = 1.0 day^−1^, and 1/*μ* = 70 years. In panel (a) two situations are shown: two and ten strains circulate within the human population. For *n* = 10, the solid blue line represents the results for *β*_3_ = *β*_4_ = 0 and the dashed red line for *β*_3_ = 0.40 day^−1^ and *β*_4_ = 0.32 day^−1^. The black line represents the threshold condition for the second strain to successfully invade and coexist with strain 1. Since empirical patterns of incidence time series are very irregular, distinguishing between chaotic and cyclical epidemics is not important so we lump them together as chaotic/cyclical epidemics and just mention them as cyclical epidemics in text. In panels (a) and (b) infectious period = 2 days and *R*_0_ = 2.0 for single infection. In panels (b) and (c) different combinations of simultaneous transmission rates of coinfections are assumed. In panel (c) the basic reproductive number of single infection (*R*_0_) varies by altering the infectious period. Panel (d) shows the interepidemic periods in years when they first emerge under the three different simultaneous transmissions of various coinfections in panel (c).

As shown above, coinfections facilitate coexistence; further they also aid the generation of sustained epidemic oscillations. Stability analysis and simulations show that with sufficiently enhanced transmissibility from coinfections the dynamic system will experience a Hopf bifurcation at which steady coexistences develop into limit cycles (Appendix).  Figure 4 shows the impact of simultaneous transmission of coinfections and antigenic diversity on the critical value of efficiency of partial transmission from coinfections (*ϕ*_*c*_) for emergence of cyclical epidemics. In the absence of simultaneous transmission of coinfections, *ϕ*_*c*_ > 1. The observation under this special situation is similar to the current view that multistrain oscillations can occur only in the case of enhancement if cross-immunity during subsequent infection is very strong [38, 62, 75]. Two previous multistrain models [59, 63] which ignored current infection concluded that as cross-immunity becomes very strong, cyclical and chaotic epidemics vanish. In the presence of simultaneous transmission of double infections (*β*_2_) and high antigenic diversity, *ϕ*_*c*_ reduces to below unit. For example, when *β*_2_ = *β*_1_/2 = 0.5,  *ϕ*_*c*_ = 0.81 for the situation of ten cocirculating strains. Comparatively, the simultaneous transmissions of triple and quadruple infections only have a much weak effect (Figures 4(b) and 4(c)). These indicate that *β*_2_ has a predominant effect on the critical value *ϕ*_*c*_. This implies that even with interference among strains, cyclic epidemics can still be generated provided there is enough simultaneous transmission of double infections and sufficient antigenic diversity.

**Figure 5:**
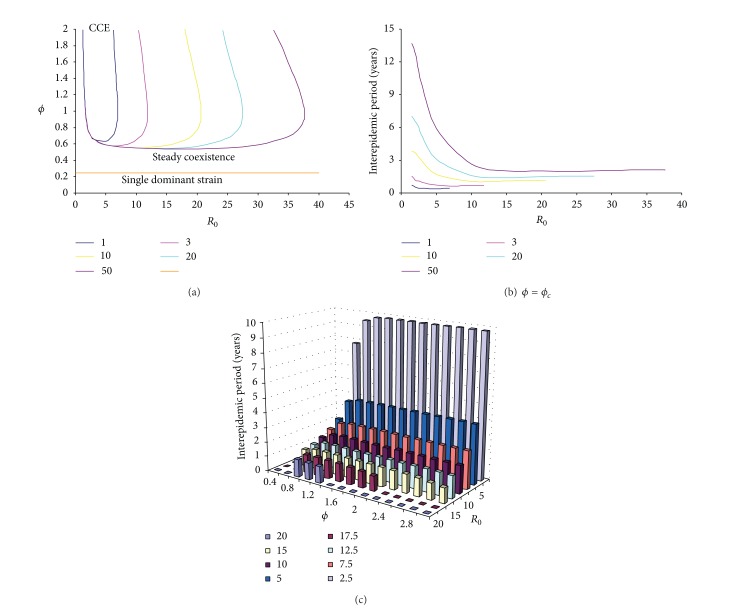
Impact of the basic reproductive number and immunity period on the critical value of the efficiency of partial transmission from coinfections (*ϕ*_*c*_) for the cyclic epidemics and their periods. Five immunity periods are illustrated: 1, 3, 10, 20, and 50 years in panels (a) and (b). Panel (a) shows the dynamical maps with CCE representing chaotic/cyclical epidemics and panel (b) shows the interepidemic period when *ϕ* = *ϕ*_*c*_. In panel (c) the interepidemic periods in years are shown against *ϕ* and *R*_0_. Other parameter values are 1/*σ* = 10 years, 1/*μ* = 70 years, *β*_1_ = 1.0 day^−1^, *β*_2_ = 0.5 day^−1^, *β*_3_ = 0.40 day^−1^, *β*_4_ = 0.32 day^−1^, *n* = 10, and *m* = 4. The basic reproductive number of single infection (*R*_0_) varies by altering the infectious period (1/*γ*).

**Figure 6:**
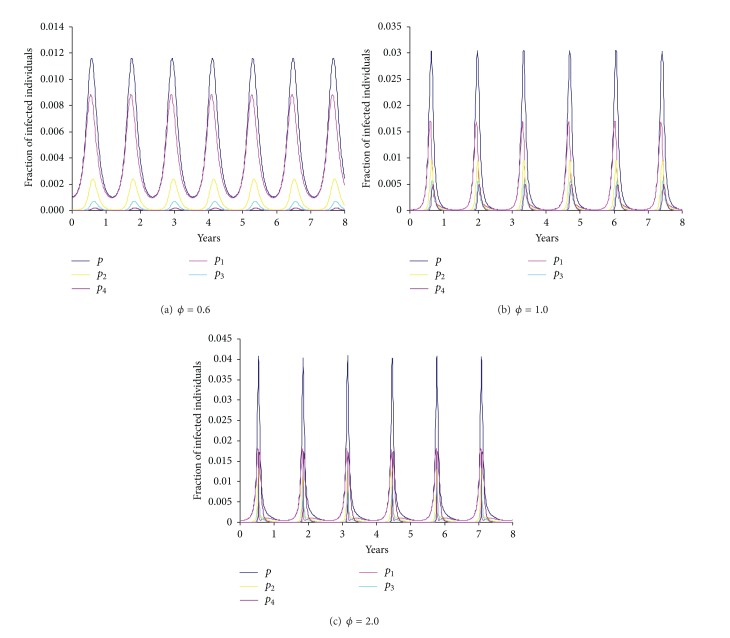
Example time series for the proportion of infected individuals. Other model parameter values are *β*_1_ = 1.0 day^−1^, *R*_0_ = 15,  1/*μ* = 70 years, *n* = 10, and *m* = 4; simultaneous transmissions of various coinfections as *β*_2_ = 0.5 day^−1^, *β*_3_ = 0.40 day^−1^, and *β*_4_ = 0.32 day^−1^. Note that the steady total prevalence from (6) would be 4.37 × 10^−3^ if the system remained in steady state. This is much smaller than the maximal prevalence within cyclical epidemics shown here.

Figure 5 shows that cyclic epidemics are also controlled by other model parameters. For a given immunity period, Figure 5(a) shows that cyclic epidemics become impossible for a too small or too large reproductive number *R*_0_. While for a given basic reproductive number, it also shows that the oscillation is prohibited under short immunity. Once emerged, the period of cyclic epidemics increases with immunity period and efficiency of partial transmission from coinfections but decreases with the basic reproductive number (Figures 5(b) and 5(c) and Figure 6). However, infectious period can only slightly increase the interepidemic period (data not shown). Figure 6 shows some example time series of cyclical epidemics under different strain interactions. When strains interfere with each other (*ϕ* < 1), single infections dominate the total prevalence; however, when they enhance each other to increase infectivity (*ϕ* > 1), the fluctuations in prevalence become large (cf.  Figure 10) and the fraction of complicated coinfections can become popular as seen in steady coexistence shown in Figure 1(d).

## 4. Discussion

In this study we investigate a multistrain SIRS epidemic model that includes coinfections and interactions between strains within coinfections. Though remaining at very low prevalence relative to single infections, coinfections and their simultaneous transmission substantially change the behaviour of infectious dynamics. They enable multiple strains to readily coexist and therefore maintain antigenic diversity. Further, interplay of strain interactions within coinfections and complete cross-immunity during subsequent infection induces sustained cyclical epidemics over a wide range of biologically realistic parameter space. In contrast to the heavily sought external seasonal forcing, this simple model provides a possible intrinsic mechanism for epidemic cycling of infectious diseases.

### 4.1. Invasion of New Strains and Antigenic Diversity

**Figure 7:**
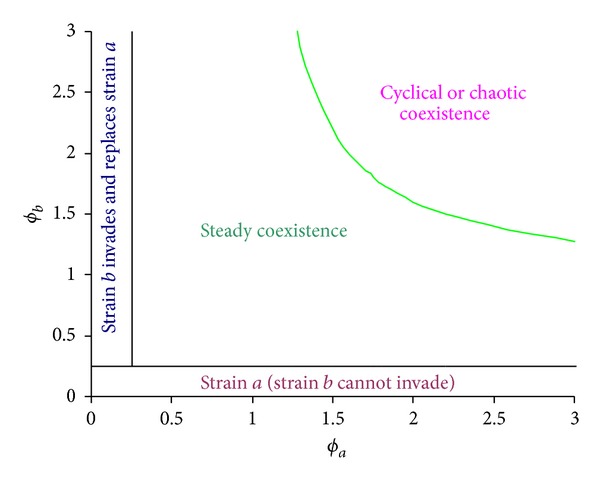
Impact of strain interactions on coexistence and sustained oscillation in incidence of infections. The host population was initially endemic with strain *a* and then challenged with strain *b*. Two strains are assumed to be equally transmissible with *β*_*a*_ = *β*_*b*_ = 0.75 day^−1^ and infectious period (1/*γ*_*a*_ = 1/*γ*_*b*_) = 2 days (i.e., *R*_0_^*a*^ = *R*_0_^*b*^ = 1.5). Coinfection can simultaneously transmit at a rate (*β*_*d*_) = 0.375 day^−1^ and with an infectious period (1/*γ*_*d*_) = 2 days. Other parameters are the immunity period (1/*σ*) = 3 years and life span (1/*μ*) = 55 years.

**Figure 8:**
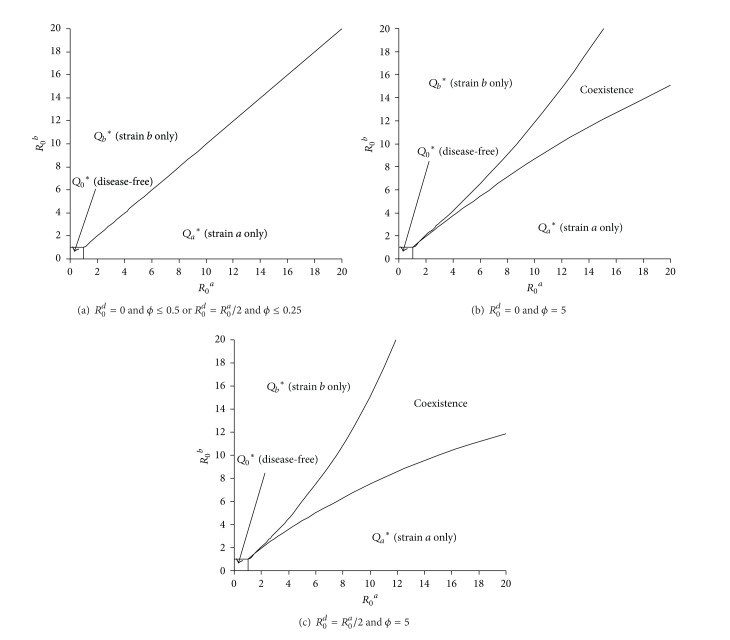
Impact of efficiency of single transmission (*ϕ*) and simultaneous transmissibility (*R*_0_^*d*^) from dual infection on the distribution of equilibria. The four equilibria on the (*R*_0_^*a*^, *R*_0_^*b*^) plane are separated by four curves: *R*_0_^*a*^ = 1, *R*_0_^*b*^ = 1, *R*_0_^*b*^(*Q*_a_*) = 1, and *R*_0_^*a*^(*Q*_b_*) = 1. In panel (a) where *R*_0_^*d*^ = 0 and *ϕ* ≤ 0.5 or *R*_0_^*d*^ = *R*_0_^*a*^/2 and *ϕ* ≤ 0.25,  *R*_0_^*b*^ > *R*_0_^*a*^ is required to maintain *R*_0_^*b*^(*Q*_a_*) ≥ 1 (and similarly requiring *R*_0_^*a*^ > *R*_0_^*b*^ to maintain *R*_0_^*a*^(*Q*_b_*) ≥ 1); hence no coexistence is possible. In panels (b) and (c) we consider the symmetric situation in which *ϕ*_*a*_ = *ϕ*_*b*_ = *ϕ* = 5 under different simultaneous transmissibility. The other parameters are infectious period (1/*γ*_*a*_ = 1/*γ*_*b*_ = 1/*γ*_*d*_) = 2 days, immunity period = 3 years, and life span = 55 years.

**Figure 10:**
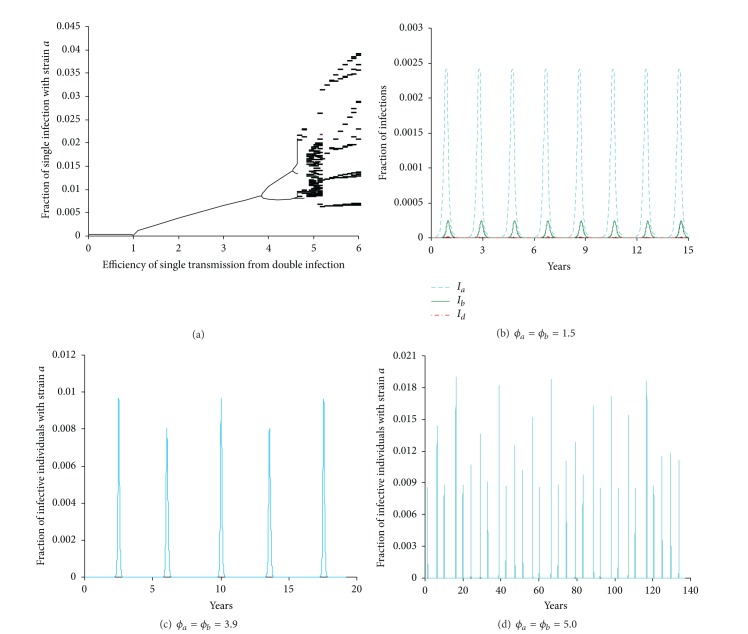
(a) Bifurcation diagram plotting the local maxima of *I*_*a*_ (the fraction of the infective individuals with strain *a*) against efficiency (*ϕ* = *ϕ*_*a*_ = *ϕ*_*b*_) of single transmission from coinfection. Numerical integration of the equations is illustrated at different values of *ϕ*_*a*_ = *ϕ*_*b*_: (b) 1.5 (period-2), (c) 3.9 (period-4), and (d) 5.0 (chaotic) for *I*_*a*_. In panel (b) the fraction of infective individuals with strain *b* and that of coinfections are also included. The other parameters are immunity period = 5 years, *β*_*a*_ = 0.75 day^−1^, *β*_*b*_ = 0.74 day^−1^, *β*_*d*_ = 0.7 day^−1^, and infectious period (1/*γ*_*a*_ = 1/*γ*_*b*_ = 1/*γ*_*d*_) = 2 days. The first 100 years were discarded. Note that as *ϕ* increases, the trough that the infection experiences at valley becomes deeper and deeper while the epidemic interval shortens from 200 to 100 days. Here the epidemic interval is defined relatively as the interval during which the fraction of infections is maintained at >10% of its peak size. This suggests that pathogen strains will suffer the trough extinction at large values of efficiency (*ϕ*).

In contrast to other coinfection models [51], we include simultaneous transmission (or cotransmission) of multiple strains within concurrent infection. Even under total cross-immunity, coinfections increase the chance of invasion by a new strain into a population at endemic with other strains (Figures 7 and 8; [40]) and simultaneous transmission enables a less transmissible strain to successfully invade (Table 4). This allows multiple strains to coexist and increases the pathogen diversity though, in its absence, only one strain would persist, as suggested in traditional competitive exclusion (Table 1; [67, 73]). This is comparable with multistrain models for dengue virus which include cross-reactive interaction during subsequent infection by other strains [76, 77]. Kooi et al. [76] show that though heterogeneity in transmission among strains reduces coexistence, the cross-reaction in second infection increases it. Interestingly, Mier-y-T-Teran-Romero et al. [77] show that combination of antibody-dependent enhancement and slight heterogeneity in transmission among strains increases persistence of four strains, suggesting evolutionary advantage of antibody-dependent enhancement. In contrast to Abu-Raddad et al. [51], our results show that, during the establishment process of a new strain, the total prevalence remains roughly unchanged. This observation suggests invisible invasion of genotypically different but antigenic indistinguishable new strains. When strains within coinfections can be transmitted simultaneously, coinfections can become prevalent if pathogen strains are highly transmissible (Figures 1, 2, and 3). However, the long immunity period and interference between strains within coinfection will reduce the opportunities of high complicated coinfection and the antigenic diversity. This is comparable with predicted patterns of antigenic diversity by other models [25].

The observed levels of antigenic diversity may be large but surely are not infinite, say, influenza A [78]. So what limits the antigenic diversity? As the total prevalence is roughly constrained by (6) when multiple strains are in steady coexistence, the fraction of each single infection decreases proportionally with the antigenic diversity. In view of the actual limited size of host populations, stochastic extinction may be one of the forces that can limit the antigenic diversity [25, 67, 74]. Further, when infections experience oscillations, trough extinction (Figure 6 and Figure 12; [65]) may be another mechanism. One important issue is that many newcomers, such as mutants, recombinants, reassortants, and immigrants, may have different epidemiological properties. Simulations show that any less transmissible newcomer will vanish no matter what the values of *β*_2_ and *ϕ* are. The newcomers will replace endemic strains once they are more transmissible, which may also reduce pathogen diversity. Because of quick introduction of newcomers, the infections that were observed may never reach a static status. These factors may work together to restrain antigenic diversity.

### 4.2. Epidemic Cycling

Though coinfection enhances coexistence of pathogen strains it alone cannot induce cyclical epidemics (Figure 5(a)). For example, Zhang et al. [36] considered a two-plant virus model in which the coinfection was included. No cyclical epidemic emerges because the infected plants die and there is no recovered compartment. The two-disease SIR models investigated by Blyuss and Kyrychko [79] and Martcheva and Pilyugin [57] included coinfections but neglected simultaneous transmission and cross-immunity. The sustained cycles were not generated in the former though induced in the latter, which is due to enhanced transmissibility of the strain that is weaker on its own.

Immunity is a common course of infection [2, 3] and is an important factor that leads to sustained oscillation [11, 15, 20]. Empirical analyses show that, for example, vaccination increases the degree of seasonal variation in the incidence of rotavirus [5]. However, theoretical investigations show that cross-immunity alone within a two-strain SIR model cannot support cyclical epidemics [63, 66, 80]. To simplify the study, we assume complete immunity and complete cross-immunity but the immunity decays. Therefore coinfection in this study is only referred to as concurrent infection (cf. [2, 38]). This is comparable with the assumption of transient strain-transcending immunity [74, 78] which states that patients who recovered from infection can be temporally protected against other strains and permanently against the previous infection strain. The decay of immunity in our model is assumed to reflect genotype changes in strains due to antigenic drift.

**Figure 11:**
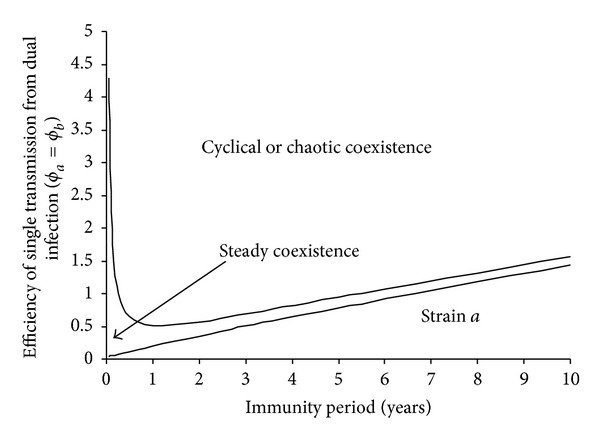
Impact of immunity period on coexistence and sustained oscillation in incidence. The narrow gap between two lines represents the area of steady coexistence. Note that when the cross-immunity remains permanent (i.e., *σ* = 0), two strains can coexist under strong enhancement in transmission (i.e., 9.1 < *ϕ*_*a*_ = *ϕ*_*b*_ < 9.2) and cyclical epidemic can emerge when *ϕ*_*a*_ = *ϕ*_*b*_ > 9.2 (data not shown). Other model parameters are *β*_*a*_ = 0.75,  *β*_*b*_ = 0.74,  *β*_*d*_ = 0.70, infectious period (1/*γ*_*a*_ = 1/*γ*_*b*_ = 1/*γ*_*d*_) = 2 days, and life span (1/*μ*) = 55 years.

In some sense the immunity period can be regarded as the “strength” of the immunity: a short immunity period implying a weak immunity. It was demonstrated here that cyclical epidemics are prohibited under weak immunity (Figure 5(a); [59]).  Figure 11 shows that pathogens that induce very short immunity can readily coexist and they remain steady if there is no immunity. Whilst those that induce lifelong immunity in host more likely lead to competitive exclusion, which suggests that extremely strong cross-immunity avoids cyclical epidemics (cf. [59, 63]). In the presence of simultaneous transmission, strain interactions within coinfections interplay with cross-immunity to generate cyclical epidemics (Figures 4 and 5). The prevalent view of intrinsic mechanisms for cyclical epidemics in the situation of strong cross-immunity is the enhancement in sequential infections between strains [38, 59, 62, 63, 75]. In contrast with this, our results reveal that, with simultaneous transmissibility of strains and antigenic diversity, cyclical epidemics could be induced even if strains within coinfections interfere with each other. Furthermore, our results show that it is the simultaneous transmission of double infections that enables this, while the simultaneous transmissibility of more highly diverse coinfections only has minor effects (Figures 4 and 5).

**Figure 12:**
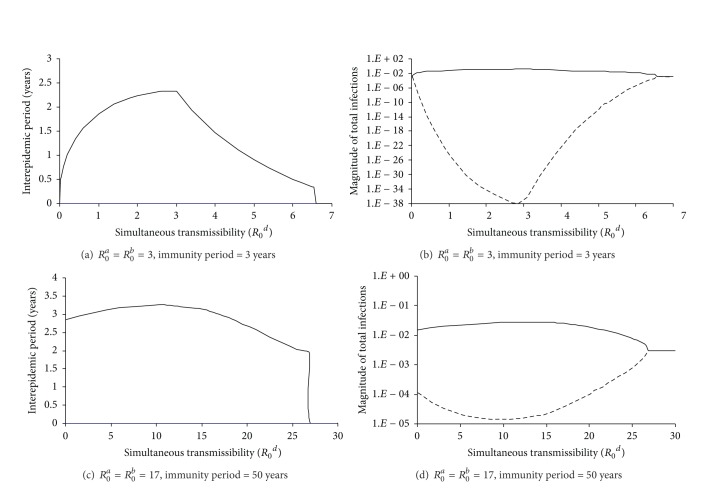
Impact of simultaneous transmissibility on the interepidemic period and amplitude of infectious transmission dynamics. Two situations of transmissibility of single pathogen infection are considered: panels (a) and (b) *R*_0_^*a*^ = *R*_0_^*b*^ = 3.0 and immunity period = 3 years, (c) and (d) *R*_0_^*a*^ = *R*_0_^*b*^ = 17 and immunity period = 50 years. In panels (b) and (d), solid lines stand for the maxima while dotted ones lined the minima of the fraction of total infections. Other parameters are the efficiency of single transmission from coinfection (*ϕ*) = 2 and the infectious period (1/*γ*_*a*_ = 1/*γ*_*b*_ = 1/*γ*_*d*_) in (a)-(b) which mimics influenza is 2 days while in (c)-(d) which mimics pertussis is 30 days. The first 100 years were discarded.

In our model, the interepidemic period is collectively determined by model parameters. Our analyses reveal that the combination of a short immunity period and a high *R*_0_ gives rise to cyclical epidemics of short periods (Figure 5). This prediction is consistent with the results of Ferguson et al. [38] and is qualitatively in agreement with the empirically periodic patterns of childhood infection dynamics: seasonal flu that has a short immunity duration of 3–8 years [12, 81] and *R*_0_ about 2 [82] was bound to annual cycles [24] while pertussis of a longer immunity period of about 30 years [83] leads to 3-4-year cycles [7]. Moreover, the interepidemic period initially increases with efficiencies of partial transmission from coinfections but quickly saturates (Figure 5(c) and Figure 6). It first increases with simultaneous transmission rate of double infections (*β*_2_) and then decreases (Figure 12) while it is insensitive to simultaneous transmission of higher diverse coinfections when epidemic cycle first emerges (Figure 4(d)). Unfortunately, we currently do not have empirical data to test these relationships.

### 4.3. Comparison to Other Models of Epidemic Cycling

Oscillations in incidence were predicted in many previous epidemic models with different underlying mechanisms, for example, in one-strain SIR models [4, 8–11], two-strain SIR models [68], and two-strain SIRS models [12] where the seasonal forcing was thought to be the mechanism. Castillo-Chavez et al. [53] established that age-structure and cross-immunity generate sustained oscillations in a two-strain SIR model while Nuño et al. [66] showed that host isolation and cross-immunity lead to periodic epidemic outbreaks. Gupta et al. [59] investigated a multistrain SIR model in which coinfections included in the force of infection to reflect the generation of new strains through recombination are not set up as separate compartments and do not contribute to transmission. This is equivalent to the situation of *ϕ* = 0 and *β*_*l*_ = 0 with *l* > 1 within our model. Gupta et al. [59] showed that cyclical and chaotic epidemic is generated at intermediate cross-immunity but disappeared at strong cross-immunity. This is in agreement with our results shown in Figure 4(a): cyclical and chaotic epidemics are prohibited when simultaneous transmission of and partial transmission from coinfections are absent. Ferguson et al. [38] and others [39, 62, 75] considered a similar SIR model and showed the enhancement of transmission during subsequent infection (i.e., so-called antibody-dependent enhancement) induces the cyclical and chaotic epidemics. Wearing and Rohani [84] and Bianco et al. [85] both consider multistrain models for dengue disease and combine antibody-dependent enhancement and temporal cross-immunity after recovering from primary infection. In contrast to Ferguson et al. [38] and other similar models, they can generate sustained oscillation in incidence even without antibody-dependent enhancement. However, the underlying mechanism for the cyclical epidemics is similar to what was shown by Nuño et al. [66]: the temporal cross-immunity class acts as the isolation class. All these dengue diseases models considered strain interaction during sequential infection, which is different from the concurrent infection we study here. Vasco et al. [61] considered a complicated variant of two-pathogen SIR model. It can produce sustained cyclical epidemics through a complicated combination of interactions between pathogens: competition due to cross-immunity, quarantine, or disease-induced mortality and cooperation due to immunosuppression and cross-enhancement. However, their model is different from ours in that the interaction between pathogens is associated with the changed susceptibility only and two pathogens cannot be simultaneously transmitted. Among these different mechanisms of epidemic cycling, which should be the truth? For example, inclusion of either age-structure [53, 57] or strain interaction within coinfection enables two-strain models to produce sustained oscillations in incidence. To discern which is correct, we need epidemiological data that include the additional information of age and antigenic diversity of infected people. These valuable data might be possible in near future, for example, for influenza viruses.

### 4.4. Simplifications and Possible Developments

Our model is simplified in several aspects. For instance, the transmission rate is assumed to be constant, which is not biologically true. The transmissibility depends on infectiousness of disease agents, contact patterns, and susceptibility of host [1, 73, 86, 87] and these factors alter with environmental conditions. It has been shown that the inclusion of a stochastic environmental transmission in a one-strain SIR model can induce periodic outbreaks of infections [15, 88]. Actually, much effort has been made to search for the seasonal forcing that was induced from the variation in contact patterns and susceptibility of host populations [4, 8, 19]. These external mechanisms are certainly important, especially for explaining the consistent peak timing of cyclical epidemics, which appears as a weakness of the model presented here. However, they have difficulty in explaining the globally quick spread of influenza [13] and especially for the cyclical dynamics of syphilis [15]. It has been suggested that the mechanisms for the common oscillation of infectious diseases must be multiple [6, 10, 19]. The true mechanism might lie in the interplay of the intrinsic (as suggested by this investigation and Grassly et al. [15]) and external periodic forcing [4, 8].

For simplicity, we assume complete cross-immunity during subsequent infection. In reality, cross-immunity is more likely to be partial [43–45, 89] and therefore infection by different strains is likely to occur sequentially. Relaxing these assumptions will improve the predictions but will not change the essential findings: coinfections facilitate pathogen diversity, and simultaneous transmission from coinfections collaborates with cross-immunity to induce cyclical epidemics.

## Figures and Tables

**Figure 9:**
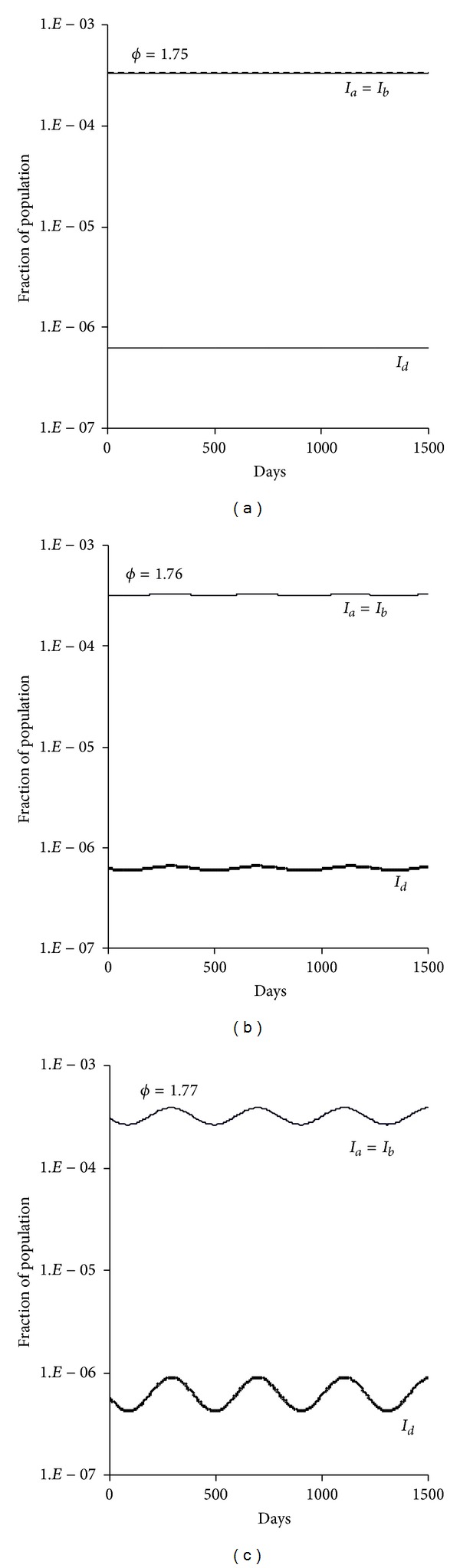
Numerical integration of (A.1) with the efficiency of single transmission from dual infection *ϕ*  ( = *ϕ*_*a*_ = *ϕ*_*b*_) = 1.75, 1.76, and 1.77. The real parts of two identical eigenvalues of characteristic equation (A.6) increase with *ϕ* and passed through 0 at 1.76 while the real parts of other two eigenvalues decrease with *ϕ* and remain negative. The imaginary part of the dominant eigenvalue is 0.0154, *T* = 410 days from (A.16), in agreement with the result shown in the graph. As we assume both strains are equal, the fractions of both single infections overlap. Other parameters are transmission rates *β*_*a*_ = *β*_*b*_ = 0.75 day^−1^, *β*_*d*_ = 0.375 day^−1^, infectious period (1/*γ*_*a*_ = 1/*γ*_*b*_ = 1/*γ*_*d*_) = 2 days, immunity period = 3 years, and life span = 55 years.

**Table 1:** The dependence of the successful invasion by an indistinguishable new strain on simultaneous transmission rates of coinfections of multiplicity *m* = 2,3, 4 and the efficiency of partial transmission from coinfections (*ϕ*). We consider the condition where a new indistinguishable strain can invade a host population at equilibrium with *n*_*e*_ endemic strains. After the challenge with a new identical strain, a new endemic will be established if condition (5) holds; otherwise, the invader is not successful and the old endemic crashes into one with a single strain. When the invasion is successful, the new endemic is established “invisibly” because the total prevalence, approximated by (6), remains unchanged. Note the prevalence of triple and quadruple infections is vanishingly small, implying their actual nonexistence. Other model parameter values are immunity period = 3 years, transmission rate of single infections (*β*_1_) = 1.0, and infectious period = 2 days; hence *R*_0_ = 2.0 for single infection.

*n*_*e*_	*β*_2_	*β*_3_	*ϕ*	*p*	*p*_1_	*p*_2_	*p*_3_	*p*_4_	*n*_ec_*
3	0.0	0.0	0.0–0.49	9.502 × 10^−4^	9.502 × 10^−4^	—	—	—	1
			0.51	9.502 × 10^−4^	9.489 × 10^−4^	1.351 × 10^−6^	1.284 × 10^−9^	6.097 × 10^−13^	4
	0.5	0.0	0.0–0.24	9.502 × 10^−4^	9.502 × 10^−4^	—	—	—	1
			0.26	9.503 × 10^−4^	9.476 × 10^−4^	2.695 × 10^−6^	3.834 × 10^−9^	3.031 × 10^−12^	4
		0.4	0.0–0.24	9.502 × 10^−4^	9.502 × 10^−4^	—	—	—	1
			0.26	9.503 × 10^−4^	9.476 × 10^−4^	2.697 × 10^−6^	6.398 × 10^−9^	5.463 × 10^−12^	4
			0.0–0.24	9.502 × 10^−4^	9.502 × 10^−4^	—	—	—	1^&^
			0.26	9.503 × 10^−4^	9.476 × 10^−4^	2.697 × 10^−6^	6.400 × 10^−9^	9.109 × 10^−12^	4^&^

2	0.0	0.0	0.0–0.49	9.502 × 10^−4^	9.502 × 10^−4^	—	—	—	1
			0.51	9.506 × 10^−4^	9.494 × 10^−4^	1.201 × 10^−6^	7.612 × 10^−10^	—	3
	0.5	0.0	0.0–0.24	9.502 × 10^−4^	9.502 × 10^−4^	—	—	—	1
			0.26	9.503 × 10^−4^	9.479 × 10^−4^	2.397 × 10^−6^	2.275 × 10^−9^	—	3
		0.4	0.0–0.24	9.502 × 10^−4^	9.502 × 10^−4^	—	—	—	1
			0.26	9.503 × 10^−4^	9.479 × 10^−4^	2.399 × 10^−6^	3.794 × 10^−9^	—	3

1	0.0	—	0.0–0.50	9.502 × 10^−4^	9.502 × 10^−4^	—	—	—	1
		—	0.51	9.502 × 10^−4^	9.493 × 10^−4^	9.020 × 10^−7^	—	—	2
		—	2	9.529 × 10^−4^	9.520 × 10^−4^	9.097 × 10^−7^	—	—	2
	0.5	—	0.0–0.25	9.502 × 10^−4^	9.502 × 10^−4^	—	—	—	1
		—	0.26	9.503 × 10^−4^	9.485 × 10^−4^	1.801 × 10^−7^	—	—	2

*n_ec_ is the number of endemic strains after the challenge of the invader.

^&^In this situation we set *β*_4_ = 0.4 while *β*_4_ = 0 for all other situations.

**Table 2:** Dependence of antigenic diversity structure in the steady coexistence on the transmission rates. Other parameter values are immunity period = 3 years, transmission rate of single infections (*β*_1_) = 1.0, and infectious period = 2 days; hence *R*_0_ = 2.0 for single infection; ten indistinguishable strains (*n* = 10) are assumed to cocirculate while three models are considered in which the most complex coinfections are of multiplicity *m* = 2,3, 4 strains, respectively.

*m*	*β*_2_	*β*_3_	*β*_4_	*ϕ*	*p*	*p*_1_	*p*_2_	*p*_3_	*p*_4_
4	0.5	0.0	0.0	0.75	9.535 × 10^−4^	9.502 × 10^−4^	3.263 × 10^−6^	7.469 × 10^−9^	1.664 × 10^−11^
		0.4	0.0	0.75	9.535 × 10^−4^	9.502 × 10^−4^	3.274 × 10^−6^	1.253 × 10^−8^	3.019 × 10^−11^
		0.4	0.4	0.75	9.535 × 10^−4^	9.502 × 10^−4^	3.274 × 10^−6^	1.256 × 10^−8^	5.038 × 10^−11^
		0.4	0.4	0.0^$^	9.487 × 10^−4^	9.455 × 10^−4^	3.214 × 10^−6^	1.214 × 10^−8^	4.826 × 10^−11^
	0.75	0.0	0.0	0.38	9.535 × 10^−4^	9.470 × 10^−4^	6.444 × 10^−6^	1.714 × 10^−8^	6.170 × 10^−11^
		0.6	0.0	0.38	9.536 × 10^−4^	9.470 × 10^−4^	6.529 × 10^−6^	4.349 × 10^−8^	1.328 × 10^−10^
		0.6	0.4	0.38	9.536 × 10^−4^	9.470 × 10^−4^	6.534 × 10^−6^	4.369 × 10^−8^	2.218 × 10^−10^
		0.6	0.4	0.0^$^	9.487 × 10^−4^	9.423 × 10^−4^	6.386 × 10^−6^	4.209 × 10^−8^	2.117 × 10^−10^

3	0.5	0.0	—	0.75	9.535 × 10^−4^	9.502 × 10^−4^	3.263 × 10^−6^	7.478 × 10^−9^	—
		0.4	—	0.75	9.535 × 10^−4^	9.502 × 10^−4^	3.274 × 10^−6^	1.249 × 10^−8^	—
		0.4	—	0.0^$^	9.486 × 10^−4^	9.454 × 10^−4^	3.213 × 10^−6^	1.216 × 10^−8^	—

2	0.5	—	—	0.75	9.535 × 10^−4^	9.502 × 10^−4^	3.256 × 10^−6^	—	—
		—	—	0.0^$^	9.486 × 10^−4^	9.454 × 10^−4^	3.223 × 10^−6^	—	—

^$^These coexistence equilibria are unstable.

**Table 3:** Four equilibria and their stability conditions of model system (A.1).

Equilibrium	Solution	Stability conditions
^ &^Disease-free (*Q*_0_*)	$\overset{-}{S}=1$, ${\overset{-}{I}}_{a}={\overset{-}{I}}_{b}={\overset{-}{I}}_{d}=\overset{-}{R}=0$	*R*_0_^a^ < 1, *R*_0_^b^ < 1, and *R*_0_^d^ < 1

^ $^Strain *a* alone (*Q*_a_*)	$\overset{-}{S}=\frac{1}{R_{0}^{a}}$, ${\overset{-}{I}}_{b}={\overset{-}{I}}_{d}=0$, ${\overset{-}{I}}_{a}=\frac{\sigma +\mu }{\sigma +\mu +\gamma_{a}}\left(1-\frac{1}{R_{0}^{a}}\right)$, $\overset{-}{R}=\frac{\gamma_{a}}{\sigma +\mu +\gamma_{a}}\left(1-\frac{1}{R_{0}^{a}}\right)$	*R*_0_^a^ > 1, *R*_0_^a^ > *R*_0_^b^, *R*_0_^a^ > *R*_0_^d^, *R*_0_^b^(*Q*_a_*) < 1

Strain *b* alone (*Q*_b_*)	$\overset{-}{S}=\frac{1}{R_{0}^{b}}$, ${\overset{-}{I}}_{a}={\overset{-}{I}}_{d}=0$, ${\overset{-}{I}}_{b}=\frac{\sigma +\mu }{\sigma +\mu +\gamma_{b}}\left(1-\frac{1}{R_{0}^{b}}\right)$, $\overset{-}{R}=\frac{\gamma_{b}}{\sigma +\mu +\gamma_{b}}\left(1-\frac{1}{R_{0}^{b}}\right)$	*R*_0_^b^ > 1, *R*_0_^b^ > *R*_0_^a^, *R*_0_^b^ > *R*_0_^d^, *R*_0_^a^(*Q*_b_*) < 1

Coexistence (*Q*_ab_*)	Interior equilibrium $\left(\overset{-}{S},{\overset{-}{I}}_{a},{\overset{-}{I}}_{b},{\overset{-}{I}}_{d},\overset{-}{R}\right)$ obtained numerically by solving equations (A.1) with the left sides being set to zero	*R*_0_^a^(*Q*_b_*) > 1, *R*_0_^b^(*Q*_a_*) > 1, and the real part of the dominant eigenvalue of Jacobian matrix of system (A.1) being negative (see Appendix A.2)

^&^ *R*_0_^*i*^, *i* = *a*, *b*, *d*, are the basic reproduction number of strain *a*, strain *b*, and dual infection, respectively, and are given by *R*_0_^i^ = β_i_/(γ_i_ + μ).

^$^ *R*_0_^b^(*Q*_a_*) represents the effective reproduction number of strain *b* upon the population in which strain *a* was established (*Q*_a_*) (see equation (A.2)).

**Table 4:** Impact of coinfection on invasion conditions.

Contribution from coinfection to transmission	Strain *b* can invade only if
&*β*_*d*_ = 0 and *ϕ*_*b*_ = 0	$R_{0}^{b}>\left(1+\frac{\left(\gamma_{a}+\mu \right){\overset{-}{I}}_{a}R_{0}^{a}}{\left(\gamma_{b}+\mu \right)}\right)R_{0}^{a}>R_{0}^{a}$

^ $^ *β*_*d*_ > 0 and *ϕ*_*b*_ = 0	*R*_0_^d^ > *R*_0_^a^

*β*_*d*_ = 0 and *ϕ*_*b*_ > 0	^%^when *R*_0_^b^ = *R*_0_^a^, $\phi_{b}>\frac{1}{2}\frac{\gamma_{d}+\mu }{\gamma_{b}+\mu }$
	^ #^when *R*_0_^b^ < *R*_0_^a^, $\phi_{b}>\frac{\gamma_{d}+\mu_{a}}{\beta_{a}{\overset{-}{I}}_{a}}\frac{1-\Gamma }{1+\Gamma }$

*β*_*d*_ > 0 and *ϕ*_*b*_ > 0	^%^when *R*_0_^b^ = *R*_0_^a^, $\frac{R_{0}^{d}}{R_{0}^{a}}+2\phi_{b}\frac{\gamma_{b}+\mu }{\gamma_{d}+\mu }>1$
	^ #^when *R*_0_^b^ < *R*_0_^a^, $\frac{R_{0}^{d}}{R_{0}^{a}}+\phi_{b}\frac{\beta_{a}{\overset{-}{I}}_{a}}{\gamma_{d}+\mu }\frac{1+\Gamma }{1-\Gamma }>1$

We consider the successful invasion conditions by investigating the scenario where the host population in which strain *a* was already established is challenged by strain *b*. In principle, it is simply that the invasion reproductive number is larger than unit (i.e., *R*_0_^b^(*Q*_a_*) > 1). In Table 4 we explore the influence of the coinfection on the successful invasion by illustrating different special situations.

^&^ ${\overset{-}{I}}_{a}$ is the fraction of infected individuals at equilibrium *Q*_a_*.

^$^Strain *a* will be replaced by the coinfection.

^#^ $\Gamma \equiv {\left(R_{0}^{b}/R_{0}^{a}\right)\left[1+\left((\gamma_{a}+\mu )/(\gamma_{b}+\mu )\right){\overset{-}{I}}_{a}R_{0}^{a}\right]}^{-1}$.

^%^The approximation under the condition $\gamma_{b}+\mu \gg \beta_{a}{\overset{-}{I}}_{a}$.

## Appendix

### A. Two-Strain SIRS Model

In this appendix we consider a special scenario with *n* = *m* = 2 but allow two strains to differ in epidemiological properties (i.e., so-called asymmetric two-strain model). From the model systems (1) and (2), the two-strain SIRS model is given by

(A.1) dSdt=μ−[βa(Ia+ϕaId)+βb(Ib+ϕbId)   +βdId+μ]S+σ(1−S−Ia−Ib−Id),dIadt=βa(Ia+ϕaId)S−βb(Ib+ϕbId)Ia−(γa+μ)Ia,dIbdt=βb(Ib+ϕbId)S−βa(Ia+ϕaId)Ib−(γb+μ)Ib,dIddt=βb(Ib+ϕbId)Ia+βa(Ia+ϕaId)Ib+βdIdS−(γd+μ)Id,

where *I*_*a*_,  *I*_*b*_, and *I*_*d*_ are the proportions in infections with strain *a* alone, strain *b* alone, and both. *β*_*a*_,  *β*_*b*_, and *β*_*d*_ are transmission rate of strain *a*, strain *b*, and both, respectively, and *γ*_*a*_,  *γ*_*b*_, and *γ*_*d*_ are their corresponding recovery rates.

There are four equilibria (see Table 3); in the following we use stability analysis to establish conditions for successful invasion and coexistence and the condition for Hopf bifurcation from steady to cyclical epidemics.

#### A.1. Invasion Conditions and Stability Analysis of Equilibria

The threshold condition for pathogen strain *a* to successfully invade a naive host population was that its basic reproductive number (*R*_0_^*a*^) must be larger than one where *R*_0_^*a*^ is defined as the average number of secondary infections that result from the introduction of a single infectious individual into an entirely susceptible population. Similarly, the critical condition for strain *b* to invade and persist in a host population in which strain *a* was established at equilibrium (*Q*_*a*_*) is its invasion (or effective) reproductive number being larger than unit; that is, *R*_0_^*b*^(*Q*_*a*_*) > 1 [57]. Applying the method of van den Driessche and Watmough [90], we obtain

(A.2) $$\begin{array}{c} R_{0}^{b}\left(Q_{a}^{*}\right)=\frac{1}{2}\left[\Phi +\sqrt{\Phi^{2}+4\Delta }\right], \end{array}$$

where

(A.3) $$\begin{array}{c} \Phi \equiv \frac{\beta_{b}}{R_{0}^{a}}\frac{1}{\gamma_{b}+\mu +\beta_{a}{\overset{-}{I}}_{a}}+\frac{\beta_{b}\phi_{b}{\overset{-}{I}}_{a}+\beta_{d}/R_{0}^{a}}{\gamma_{d}+\mu }, \end{array}$$

(A.4) $$\begin{array}{c} \Delta \equiv \frac{\beta_{b}}{R_{0}^{a}}\frac{\phi_{b}\beta_{a}{\overset{-}{I}}_{a}-\beta_{d}/R_{0}^{a}}{\left(\gamma_{d}+\mu \right)\left(\gamma_{b}+\mu +\beta_{a}{\overset{-}{I}}_{a}\right)}. \end{array}$$

In expressions (A.3)-(A.4), ${\overset{-}{I}}_{a}$ is the fraction of infective individuals with strain *a* at equilibrium *Q*_*a*_*;  *R*_0_^*d*^ represents the basic reproductive number of double infection. It is worth mentioning that *R*_0_^*b*^(*Q*_*a*_*) = 1 when Φ + Δ = 1; thus, when Φ + Δ > 1,  *R*_0_^*b*^(*Q*_*a*_*) > 1. To demonstrate how the interaction between strains determines the successful invasion and coexistence, we rearrange the inequality Φ + Δ > 1 into

(A.5) $$\begin{array}{c} \beta_{d}>\left[\left(\gamma_{d}+\mu \right)-\phi_{b}\beta_{a}{\overset{-}{I}}_{a}\frac{1+\Gamma }{1-\Gamma }\right]R_{0}^{a} \end{array}$$

with $\Gamma \equiv \left(R_{0}^{b}/R_{0}^{a}\right){\left(1+({\overset{-}{I}}_{a}\beta_{a}/\left(\gamma_{b}+\mu \right))\right)}^{-1}$. Under the situation where *R*_0_^*a*^ = *R*_0_^*b*^ and $\gamma_{b}+\mu \gg \beta_{a}{\overset{-}{I}}_{a}$ which equivalently requires that the infectious period is much shorter than the duration of immunity and life span, we have $\Gamma \approx 1-{\overset{-}{I}}_{a}\beta_{a}/(\gamma_{b}+\mu )$, and the above inequality is approximated as follows:

(A.6) $$\begin{array}{c} \beta_{d}>\left[\left(\gamma_{d}+\mu \right)-2\phi_{b}\left(\gamma_{b}+\mu \right)\right]R_{0}^{a}. \end{array}$$

By the symmetry between strain *a* and strain *b*, *R*_0_^*a*^(*Q*_*b*_*) can be obtained by swapping the indexes *a* and *b* in the above equations. When *R*_0_^*a*^(*Q*_*b*_*) > 1 and *R*_0_^*b*^(*Q*_*a*_*) > 1 hold simultaneously, two strains coexist. To illustrate these relationships, two examples are shown: one for how strain interaction affects the invasion and coexistence (Figure 7) and the other for distribution of four equilibria and the effect of simultaneous transmission (Figure 8).

The stability conditions for equilibria can be obtained by analysing the eigenvalues of Jacobian matrix of the system (A.1) at equilibria. Consider the stability condition of equilibrium *Q*_*a*_* of single infection with strain *a*; its characteristic equation can be reduced to

(A.7) |M−λI| =|−βaI−a−μ−σ−λ−βaS−−σβaI−a−λ|  ×|βbS−−βaI−a−γb−μ−λϕbβbS−(βa+βb)I−aϕbβbI−a+βdS−−γd−μ−λ| ≡|C|×|D|=0.

Here $\overset{-}{S}$ and ${\overset{-}{I}}_{a}$ are given in Table 3. Matrix** C** always has two negative eigenvalues while matrix** D** has negative eigenvalues only if the following inequalities hold:

(A.8) $$\begin{array}{c} \beta_{b}\left(\frac{1}{R_{0}^{b}}-\frac{1}{R_{0}^{a}}\right)+\beta_{d}\left(\frac{1}{R_{0}^{d}}-\frac{1}{R_{0}^{a}}\right)>-\left(\beta_{a}-\beta_{b}\phi_{b}\right){\overset{-}{I}}_{a}, \end{array}$$

(A.9) 1>βbS−γb+μ+βaI−a+βbϕbI−a+βdS−γd+μ+(βaϕbI−a−βdS−)(γb+μ+βaI−a)(γd+μ)βbR0a≡Φ+Δ.

Inequality (A.9) is equivalent to *R*_0_^*b*^(*Q*_*a*_*) < 1 and it can also be expressed as follows:

(A.10) R0a>R0b(1−R0dR0a)γb+μγb+μ+βaI−a+R0d+R01βbϕbI−aγd+μ(1+γa+μγb+μ+βaI−a).

Combining this with inequality (A.8), we have *R*_0_^*a*^ > 1,  *R*_0_^*a*^ > *R*_0_^*b*^, and *R*_0_^*a*^ > *R*_0_^*d*^. These are stability conditions listed in Table 3. Similarly we can obtain the stability conditions of equilibria *Q*_*b*_* and *Q*_0_*. Due to the complexity, the stability condition for coexistence equilibrium *Q*_*ab*_* is hard to obtain analytically though in principle it can be determined through the negativeness of the eigenvalues of the characteristic equation (see below). Interestingly, numerical simulations (data not shown) demonstrate that even when *R*_0_^*a*^ < 1 and *R*_0_^*b*^ < 1, conditions that *R*_0_^*d*^ > 1 plus *ϕ*_*a*_ > 0 and *ϕ*_*b*_ > 0 can guarantee the coexistence of both strains (cf. [90]).

Some special situations are listed in Table 4 to illustrate the effect of coinfections on the successful invasion. Without simultaneous transmission of coinfections, strain *b* can invade only when it has a higher basic reproductive number *R*_0_^*b*^ (see also Figure 8). However, strain interactions within coinfections change this outcome. If *R*_0_^*b*^ = *R*_0_^*a*^, strain *b* can invade if the efficiency of transmitting strain *b* from coinfection (*ϕ*_*b*_) is larger than (1/2)(1/*γ*_*b*_)/(1/*γ*_*d*_), suggesting that if coinfections have doubled infectious periods, strain *b* can invade if *ϕ*_*b*_ > 25%. On the other extreme scenario where *ϕ*_*b*_ is nil, strain *b* can invade only when *R*_0_^*d*^ > *R*_0_^*a*^ and the previous single infection will be replaced by coinfections. In particular, it is possible for a less transmissible strain *b* (i.e., *R*_0_^*b*^ < *R*_0_^*a*^) to invade if both *β*_*d*_ and *ϕ*_*b*_ are strong enough.

Next we consider a symmetric situation where two strains share the same properties: *β* ≡ *β*_*a*_ = *β*_*b*_,  *γ* ≡ *γ*_*a*_ = *γ*_*b*_ = *γ*_*d*_ and *ϕ* ≡ *ϕ*_*a*_ = *ϕ*_*b*_ so that from (A.1) we have at equilibrium ${\overset{-}{I}}_{1}={\overset{-}{I}}_{2}$ and

(A.11) $$\begin{array}{c} {\overset{-}{I}}_{1}+{\overset{-}{I}}_{2}+{\overset{-}{I}}_{d}=\frac{\sigma +\mu }{\sigma +\mu +\gamma }\left(1-\overset{-}{S}\right). \end{array}$$

When *ϕ* is not very strong and *β* > *β*_*d*_ so that only the steady coexistence is possible, numerical results show that $\overset{-}{S}\gg {\overset{-}{I}}_{1}={\overset{-}{I}}_{2}\gg I_{d}$ (Tables 1 and 2); from the second and third equations of (A.1), we obtain the approximation

(A.12) $$\begin{array}{c} \overset{-}{S}\approx \frac{\left(\mu +\gamma \right)}{\beta }=\frac{1}{R_{0}^{a}}=\frac{1}{R_{0}^{b}}. \end{array}$$

Putting (A.12) into (A.11) gives rise to approximation (6) in the text for the situation of two symmetric strains.

#### A.2. Analysis of Hopf Bifurcation

Because of the complexity in model system (A.1), the analytical expression for the condition of Hopf bifurcation is not obtained. Taking the advantage of R computing language (http://www.r-project.org/), however, we can numerically test and obtain the condition under which the Hopf bifurcation occurs. The characteristic equation at the coexistence equilibrium (*Q*_*ab*_*) is as follows:

(A.13) $$\begin{array}{c} \left|M-\lambda I\right|=\left|\begin{array}{cccc} m_{11}-\lambda & m_{21} & m_{31} & m_{41}\\ m_{12} & m_{22}-\lambda & m_{32} & m_{42}\\ m_{13} & m_{23} & m_{33}-\lambda & m_{43}\\ m_{14} & m_{24} & m_{34} & m_{44}-\lambda \end{array}\right|=0, \end{array}$$

where elements *m*_*ij*_ are functions of the interior equilibrium $\left(\overset{-}{S},{\overset{-}{I}}_{a},{\overset{-}{I}}_{b},{\overset{-}{I}}_{d},\overset{-}{R}\right)$ and model parameters. The characteristic equation can be written as follows:

(A.14) $$\begin{array}{c} \lambda^{4}+a_{3}\lambda^{3}+a_{2}\lambda^{2}+a_{1}\lambda +a_{0}=0. \end{array}$$

The coefficients *a*_*k*_,  *k* = 0,1,…, 3, are the functions of matrix elements *m*_*ij*_,  *i*, *j* = 1,…, 4.

The quartic equation (A.14) can be solved by Ferrari's method. To investigate the Hopf bifurcation from a steady state to a periodic cycle in incidence, we examine the eigenvalues against the efficiency (*ϕ*_*a*_, *ϕ*_*b*_) of single transmission from the coinfection. In general, the four eigenvalues can be expressed as follows:

(A.15) λj(ϕa,ϕb)=ηj(ϕa,ϕb)+iωj(ϕa,ϕb),             j=1,…,4.

When the real parts *η*_*j*_ are negative, the steady coexistence equilibrium is stable. However, when values of (*ϕ*_*a*_, *ϕ*_*b*_) increase to some critical values (*ϕ*_*a*0_, *ϕ*_*b*0_), real part *η*_*j*_ of some eigenvalues will increase to zero and the equilibrium loses its stability and a Hopf bifurcation occurs [91], from which oscillation in incidence will be induced. The imaginary part of the dominant eigenvalue, $\hat{\omega }(\phi_{a},\phi_{b})$, is the frequency of the oscillation when the real part $\hat{\eta }(\phi_{a},\phi_{b})$ passes through zero to become positive and the interepidemic period can be calculated as follows:

(A.16) $$\begin{array}{c} T=\frac{2\pi }{\hat{\omega }\left(\phi_{a},\phi_{b}\right)}. \end{array}$$

One numerical example was shown in Figure 9. As the cooperation between strains within coinfections becomes strong enough, complicated dynamical behaviour such as period-doubling bifurcation and chaotic epidemics can be generated (Figure 10). Similarly, this can also be tested against other model parameters.

Interestingly, the fraction of infections at the peak of a cyclical epidemic is much larger than that at equilibrium of a single strain established alone. As illustrated in Figure 10(b), the peak fraction of single infections with dominant strain *a* is 0.24% while it is 0.04% at equilibrium if it alone infects the host population (from Table 1 and see also Figure 10(a)). Therefore we should be cautious when explaining the meanings and estimating values of the basic reproductive number (*R*_0_) for cyclical infections. Moreover, the fraction of single infection with a slightly less transmissible strain is much lower while the fraction of coinfections remains extremely low even at the peak. In Figure 10(b), they are 0.024% and 0.001%, respectively, which indicates the chance for coinfections to be detected being 1/264 ≈ 0.4% in relation to single infections. Therefore it is understandable that coinfections are practically hard to attract sensible attention, especially if advantageous technology such as genotype typing is not readily available.

The cyclical epidemics become hard to generate when immunity period becomes either too short or too long (Figure 11). Given low reproductive numbers for two different strains but relatively high transmissibility for double infection as in Figure 11, the critical efficiency of single transmission from double infection for cyclical epidemics is *ϕ*_*a*0_ = *ϕ*_*b*0_ = 4.0 for immunity that lasts for one month or *ϕ*_*a*0_ = *ϕ*_*b*0_ = 9.2 for lifelong immunity. This may be too high for most pathogens. However, when the duration of immunity takes some intermediate values, a low efficiency of single transmission from double infection should be sufficient to maintain the cyclical epidemics. When oscillation is induced, the interepidemic period increases with the efficiency (*ϕ*) of single transmission from coinfections (cf. Figures 10(b) and 10(c)). While it first increases with the simultaneous transmissibility (*R*_0_^*d*^), after reaching peak at a critical value, it decreases (Figure 12).
